# Illuminating the Patterns of Fungal Community Succession, Physicochemical Properties, Volatiles and Their Relationships in Fermented Grains for the Production of Chinese Strong-Flavor Baijiu

**DOI:** 10.3390/foods15030418

**Published:** 2026-01-23

**Authors:** Yaping Wang, Yitong Zhao, Rongyu Du, Danyang Fu, Mingdong Deng, Hua Li, Famou Guo, Zhaoxiang Wang, Xiaolong Hu

**Affiliations:** College of Food and Bioengineering, Zhengzhou University of Light Industry, Zhengzhou 450001, China; 2021015@zzuli.edu.cn (Y.W.); 332303060294@zzuli.edu.cn (Y.Z.); drongyu_0830@163.com (R.D.); fdy2426026280@163.com (D.F.); 332103020200@zzuli.edu.cn (M.D.); hnyslh@163.com (H.L.); guofamou@zzuli.edu.cn (F.G.)

**Keywords:** Chinese strong-flavor Baijiu, fungal community succession, influencing factors, volatile compounds, high-throughput sequencing

## Abstract

Fermented grains (FGs) for Chinese strong-flavor Baijiu (CSFB) serve as both microbial habitats and flavor sources, yet the correlations among fungal communities, physicochemical properties, and volatiles during long-term fermentation remain insufficiently understood. To address this gap, this study employed Illumina HiSeq high-throughput sequencing, physicochemical analysis, and GC-MS for systematic investigation. Fermentation was divided into early, middle, and late stages based on FGs’ physicochemical dynamics and eukaryotic microbial diversity. A total of 9 fungal phyla and 195 genera were detected, with 12 dominant genera (e.g., *Thermoascus*, *Aspergillus*, *Kazachstania*). Forty-seven volatiles were identified, showing increasing diversity and richness. Redundancy Analysis revealed total acids exerted the most significant effect on dominant fungal succession, while network analysis screened 10 key genera (e.g., *Mortierella*, *Trichoderma*) pivotal for community structure. Additionally, *Trichoderma*, *Fusarium* and other genera correlated with important flavors like 1-butanol and 1-hexanol. This study clarifies the complex interactions in FGs, provides theoretical support for CSFB quality improvement via biofortification or environmental control, and offers a reference for revealing the ecological mechanisms underlying FG microbial community assembly.

## 1. Introduction

CSFB is one of the most mainstream flavor types of Chinese Baijiu. Produced mainly from sorghum through core technologies including pit mud fermentation, successive grain residue blending, and mixed steaming–distillation, CSFB exhibits distinct sensory attributes: intense pit aroma, mellow-sweet and clean taste, harmonious flavor profile, and long-lasting aftertaste. It has gained great popularity among consumers worldwide [1]. During the long-term solid-state fermentation process for CSFB production, FGs act not only as substrates for microbial metabolism but also as direct sources of flavor compounds [2]. For example, yeast genera (e.g., *Saccharomyces cerevisiae*, *Pichia*, *Hansenula* and others) can efficiently convert carbohydrates into alcohol and produce various flavor substances such as higher alcohols and esters during metabolism, endowing the Baijiu with a unique aroma and mellow taste. *Aspergillus* genera (e.g., *Aspergillus oryzae*, *Aspergillus niger* and others), as another important group of fungi in FGs, can secrete a variety of enzymes including amylase and glucoamylase. These enzymes decompose polysaccharides such as starch in FGs into monosaccharides like glucose, which not only provides substrates for yeast fermentation but also generates flavor substances [3,4]. Thus, the complex fungal communities in FGs produce compounds such as esters, acids, aldehydes, higher alcohols, ketones, phenols, and pyrazines through glucose metabolism during the solid-state fermentation of Baijiu, thereby imparting CSFB with distinct flavor characteristics. Therefore, deciphering the structure and diversity of eukaryotic microbial communities in FGs during Baijiu brewing has become one of the research hotspots in recent years [5,6].

Relying on technologies such as high-throughput sequencing and microbiomics, the diversity and succession patterns of prokaryotic microbial communities in CSFB have been relatively fully elucidated. It has been reported that the CSFB fermentation process can be divided into two stages according to the temperature of FGs. The abundance of prokaryotic microorganisms in FGs is relatively high in the initial stage, then decreases significantly, and finally stabilizes. The changes in prokaryotic microbial communities interact with the physicochemical properties of FGs [6]. Huang et al. indicated that moisture content, acidity, and starch are the most significant physicochemical indicators affecting the evolution of bacterial community structure in strong-flavor FGs, while reducing sugars have a minor impact on bacterial communities at different fermentation stages. Moisture content is significantly positively correlated with *Lactobacillus*, but significantly negatively correlated with *Bacillus*, *Staphylococcus*, *Thermoactinomyces*, and other genera [7,8]. Current studies generally hold that during the long-term solid-state fermentation of CSFB, the relative abundance and functional roles of different prokaryotic microbial species change over time. These changes are not only affected by physicochemical properties but also regulated by intrinsic microbial interactions [9,10]. As the carrier of microbial metabolism and the core site for product transformation, the dynamic changes in the microbial community structure of fermented FGs interact with and are mutually causal to physicochemical properties such as moisture content, acidity, reducing sugar content, alcohol concentration, and starch content. Together, they determine the fermentation efficiency of FGs, the production of flavor compounds, and the final quality of Baijiu [6,11]. Notably, although there is an association between changes in the physicochemical properties of FGs and shifts in microbial communities, current reports on the correlation between physicochemical parameters of FGs and fungal microbial communities during the long-cycle fermentation of CSFB remain scarce. Therefore, in-depth research on the physicochemical and fungal-related factors affecting the fungal microbial communities of CSFB-FGs will help reveal the potential mechanisms underlying the dynamic changes in microbial communities in the FG ecosystem.

As the core substrate for fermentation, the FGs of CSFB harbor a complex ecosystem composed of microbial groups such as bacteria, fungi, and Actinomycetes. As mentioned previously, this microbial community exhibits distinct spatiotemporal dynamic variation characteristics; however, the correlations among microbial communities and between microbial communities and volatile compounds remain unclear. Currently, it is difficult or even impossible to reveal the complex relationships between microbial communities in FGs and volatile compounds using culture-dependent methods, as these methods are not applicable to some unculturable microorganisms present in samples [12]. In recent years, it has become possible to identify correlations with the aid of multivariate statistical analyses (e.g., network analysis, redundancy analysis). For example, in the production of CSFB, the genera *Thermus*, *Rhizomucor* and *Torulaspora* show a strong correlation with ethyl lactate [2]; the genera *Thermus* and *Saccharomyces* are positively correlated with alcohols, while *Thermus* alone is positively correlated with acids; 2,3-butanediol is significantly positively correlated with the presence of *Saccharomyces*, *Aspergillus*, *Millerozyma*, *Torulaspora* and *Wickerhamomyces* [6]. There are few reports on the correlations between fungal microorganisms and flavor substances in FGs of CSFB Therefore, it is necessary to further explore the potential correlations between fungal communities and flavor compounds in the complex FG ecosystem of CSFB, which can provide a theoretical basis for improving the quality of CSFB through microbial enhancement technology.

Collectively, the correlations between physicochemical parameters and fungal communities, the intra-interaction network characteristics of eukaryotic microbial communities, as well as the specific correlation patterns between fungi and key flavor compounds (e.g., higher alcohols and esters) during the long-term fermentation of CSFB remain largely elusive. In this study, by combining physicochemical analysis, gas chromatography-mass spectrometry (GC-MS) detection, and Illumina HiSeq sequencing technology, we elucidated the dynamic variation characteristics of physicochemical properties, eukaryotic microbial communities, and flavor compounds in FGs at different time points (0, 7, 15, 25, 45, and 60 d) during CSFB fermentation. Furthermore, we revealed the correlation relationships between key eukaryotic taxa, physicochemical factors, and flavor substances, and constructed the intra-interaction network of eukaryotic microorganisms. The results of this study can provide a theoretical basis for improving the quality stability of CSFB and optimizing the fermentation process.

## 2. Materials and Methods

### 2.1. Sample Collection

Two parallel pit cellars were randomly selected from a well-known local CSFB company (Shanmenxia City, China) for follow-up sampling on days 0, 3, 7, 15, 25, 45, and 60 of the fermentation process. The lower layer of each pit, approximately 50 cm from the bottom, was sampled with 50 g of FGs collected at three diagonal positions, each 20 cm away from the pit wall. A total of 21 samples were obtained by mixing these collections, representing the FG-samples. These 21 samples were then immediately transferred into sterile bags and stored at −30 °C for further analysis.

### 2.2. Determination of Physicochemical Properties of FGs

The moisture content of FG was determined by drying at 105 °C in an oven until a constant weight was reached. pH value was determined by potentiometric method. Total acid content (TA) was determined by acid-base neutralization titration method. The content of NH_4_^+^ was determined according to the method described by Shen [13]. Ethanol content (Et), reducing sugars content (RS) and total esters content (TE) were determined with reference to the literature [14].

### 2.3. Treatment of FG Samples

According to the previous method, the FGs were pretreated as follows: 5 g of FGs were taken and 1% CaCl_2_ (Aladdin, Shanghai, China) was added. The mixture was then combined with 10 mL of ultra-pure water and soaked overnight at 4 °C. Afterward, the sample was ultrasonicated in an ice water bath for 30 min (KQ-500DE, Kunshan Ultrasonic Instrument Co., Ltd., Kunshan, China) [15]. Subsequently, the soaking liquid was centrifuged at 6000 rpm at 4 °C for 20 min (5810R, Eppendorf, Shanghai, China), and 2 mL of the supernatant was transferred to a new 10 mL centrifuge tube. To this, 2 mL of dichloromethane (Aladdin, Shanghai, China) and 5 μL of an internal standard solution (consisting of 1% tert-amyl alcohol, 2-ethylbutyric acid, and n-amyl acetate, chromatographic grade, purchased from Solarbio Co., Ltd., Beijing, China) were added for extraction. Following vortex oscillation at maximum speed for 1 min, the mixture was allowed to separate into layers for 1–2 min. The lower organic phase (1 mL) was aspirated using a disposable sterile syringe (Kangjian Medical Supplies Co., Ltd., Taizhou, China), filtered through a 0.22 μm organic filter membrane (Millipore Sigma, Shanghai, China), and injected into a sample vial for analysis by GC-MS (7890B-5977A, Agilent Technologies, Santa Clara, CA, USA).

### 2.4. GC-MS Analysis Conditions

The chromatographic column used was a DB-FFAP (30 m × 0.25 mm × 0.25 μm) capillary column (Agilent Technologies, Santa Clara, CA, USA), and the temperature program was set as follows: the initial temperature was maintained at 45 °C for 3 min, then the temperature was increased to 220 °C at a rate of 5 °C/min and held for 5 min, and finally, the temperature was increased to 250 °C and held for 2 min. The inlet temperature was set at 250 °C; the pre-column pressure was 7.361 psi; the carrier gas was high-purity He (99.999%, Linde Gas (China) Co., Ltd., Shanghai, China); the carrier gas flow rate was 1.0 mL/min; the mode was set to non-shunt; the solvent delay time was 3.6 min; and the injection volume was 1 μL. The ion source was an EI source maintained at 230 °C, the quadrupole temperature was 150 °C, and the scanning range was 35–550 amu.

### 2.5. Semi-Quantitative Analysis of Volatile Compounds in FGs

The characteristic ions of the volatile compounds were identified based on their mass spectral characteristics (*m*/*z* 60), and the substances corresponding to each peak were then identified by searching the GC-MS standard mass spectral database. Finally, the ratio of the peak area of each compound to the internal standard peak area was used as a semi-quantitative result.

### 2.6. Extraction, Amplification and Sequencing of DNA from FG-Samples

DNA from FGs was extracted following the instructions of the Magen Hi Pure Soil DNA Kit (Magen Biotechnology Co., Ltd., Guangzhou, China). The quality of the extracted DNA was then assessed using 1% agarose gel electrophoresis (Aladdin, Shanghai, China). Total DNA samples that passed the quality assessment were amplified using primers ITS1F (5′-CTTGGTCATTTAGAGGAAGTAA-3′) and ITS2(5′-GCTGCGTTCTTCATCGATGC-3′) (Sangon Biotech (Shanghai) Co., Ltd., Shanghai, China) [16]. The total DNA samples were prepared and purified according to previous methods for PCR amplicons, incorporating sample-specific barcodes [17]. The concentration of the DNA was determined using a Thermo Scientific NanoDrop 2000 UV-Vis spectrophotometer (Thermo Fisher Scientific, Waltham, MA, USA). Following this, the purified PCR products were used to construct PCR amplicon libraries. Libraries that passed the quality check were sequenced using the Illumina HiSeq2500 platform (Illumina, San Diego, CA, USA).

### 2.7. Data Processing

Firstly, the raw reads obtained from sequencing were filtered using Trimmomatic v0.33 software [18]. Then, cutadapt 1.9.1 software was used to identify and remove primer sequences, resulting in high-quality reads free of primer sequences. Subsequently, FLASH v1.2.7 software was employed to splice the high-quality reads from each sample based on overlap. This splicing process resulted in clean reads. Following this, UCHIME v4.2 software was applied to identify and eliminate chimeric sequences, yielding the final effective reads [19].

For data analysis, Microsoft Excel 2019 was used. Histograms, principal component analysis, and heat maps were created using Origin 2021, while redundancy analysis was performed using Canoco5 software. Network analysis was conducted to assess fungal community correlation, with genera selected as nodes of the network. Only genera with an average relative abundance of more than 0.1% were included. To ensure significant and strong relationships between edges, only pairwise correlations with Spearman’s |*ρ*| > 0.7 and *p* < 0.01 were selected. The network properties were then visualized. The results were visualized using the Gephi interactive platform (Version 0.10). Finally, Cytoscape (Version 3.10.0) software was utilized to construct the fungal and volatiles network diagrams [20].

### 2.8. Data Availability

The sequencing data were submitted to the Sequence Read Archive (SRA) of the NCBI database under BioProject PRJNA1065102 (BioSample accession numbers SUB14153192).

## 3. Results

### 3.1. Dynamic Changes in the Physicochemical Properties of FGs During Fermentation

**Figure 1:**
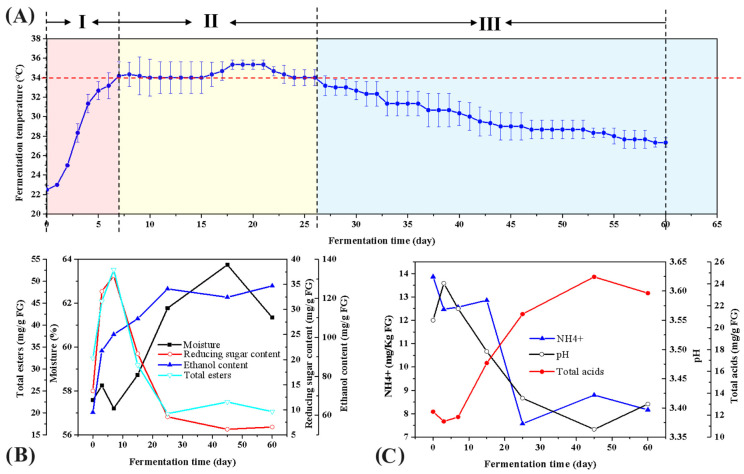
Dynamic changes in physicochemical indexes in the fermentation process of FGs. Curves of temperature change (**A**), and physicochemical factors (**B**,**C**).

The entire fermentation process can be divided into three distinct stages based on the dynamic changes in the fermentation temperature. A thorough analysis of the physicochemical properties of the FGs during the fermentation process was conducted. The temperature in FGs exhibits a trend of first increasing and then decreasing (Figure 1). The results indicate that during the initial stage of fermentation (spanning 0 to 7 days), the pH value the moisture, TE in FGs initially increased and then decreased, while RS, Et, and TA increased with fermentation progress. Conversely, the content of NH_4_^+^ showed an opposite trend. During the second stage of the fermentation process (7–25 days), the content of moisture, Et, and TA continued to increase, while RS, pH value, and TE showed an opposite trend. The content of NH_4_^+^ initially increased and then decreased. In the third stage of fermentation (45–60 days), the contents of Et and pH value decreased initially and then increased, while moisture, TA, and TE showed a reverse trend. The RS continued to increase, and the NH4^+^ content continued to decrease. In the second and third stages of the fermentation process (7–60 days), the RS exhibited an inverse relationship with the change in Et, while TA displayed an inverse relationship with the change in pH value.

The temperature of FGs exhibited a trend of first increasing and then decreasing (Figure 1A). Meanwhile, this study systematically analyzed the physicochemical properties of FGs during the fermentation process (Figure 1B,C). As can be seen from Figure 1A, the temperature of FGs generally showed a trend of increase, stabilization and decrease. In the early stage of fermentation, the temperature rose rapidly; starting from the 7th day, the temperature increased gently; and starting from day 25, the temperature began to show a slow downward trend. Among them, day 7 and day 25 were key nodes for temperature changes. Combined with the changes in physicochemical properties shown in Figure 1B,C, the entire fermentation process was finally divided into three stages, namely the early stage (0–7 d), the middle stage (7–25 d) and the late stage (45–60 d). It is speculated that the rapid temperature rise in the early fermentation stage may be attributed to the rapid growth and reproduction of microorganisms, which produce a large amount of heat while decomposing organic matter. As can be seen from the determination results of physicochemical properties in Figure 1B,C, in the early stage of fermentation, the pH value, moisture and TE of FGs all showed a trend of first increasing and then decreasing; the RS, Et and TA gradually increased with the fermentation process, while the NH_4_^+^ content showed the opposite trend. In the middle stage of fermentation, the contents of moisture, Et and TA continued to increase; the RS, pH value and TE showed a downward trend; and the NH_4_^+^ content first increased and then decreased. In the late stage of fermentation, the Et and pH value first decreased and then increased; the moisture, TA and TE showed the opposite trend; the RS continued to increase; and the NH_4_^+^ content continued to decrease. From the middle to the late stage of fermentation (7–60 d), the change in RS content was negatively correlated with that of Et content, and the change in TA content was negatively correlated with that of pH value.

### 3.2. Dynamics of Volatile Compounds in FGs

**Figure 2:**
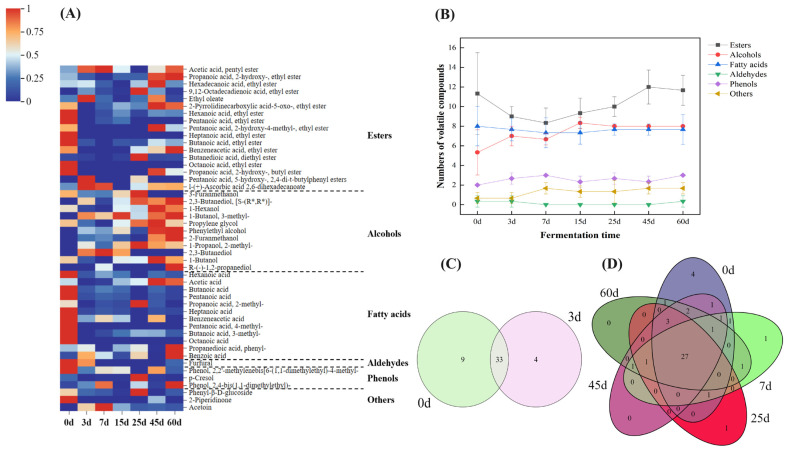
Changes in the contents and numbers of the volatile compounds of FGs at different time-points during CSFB fermentation. Heat map of 74 volatile compounds in FGs at different fermentation periods (**A**). Numbers of volatiles in FGs at different fermentation stages (**B**). Venn analysis showed the distribution of volatiles in FGs when fermented day 0 and day 3 (**C**) and in the samples from pre-fermentation (0–3 days), mid-fermentation period (7–25 days) and late-fermentation period (45–60 days) stages (**D**).

A total of 47 volatile compounds were identified in the FG samples, including 17 esters, 11 alcohols, 12 fatty acids, 1 aldehyde, 3 phenols, and 3 other volatile compounds (Figure 2A).

Overall, the contents of various volatile compounds changed notably during the fermentation process. Among them, 20 volatile compounds showed significant differences at different fermentation time points, including 10 esters, 5 phenols, 4 alcohols, and 1 other substance. For example, 2-ethyl-2-methyltridecanol, heptanoic acid, 2-(3-acetoxy-4,4,14-trimethylandrost-8-en-17-yl) propanoic acid, and furfural were present in high contents in the samples from the early fermentation stage; ethyl valerate, 2-octyl succinate, 3-tetradecyl methoxyacetate, 4-tetradecyl methoxyacetate, (S)-(+)-1,2-propanediol, and n-hexadecanoic acid showed increased contents in the samples from the middle fermentation stage; while various esters such as ethyl 2-hydroxy-4-methylpentanoate, butyl 2-hydroxypropionate, and ethyl (S)-2-hydroxypropionate were present in high contents in the samples from the late fermentation stage. During the fermentation process, the total number of volatile compounds and the number of alcohols in the FG samples increased markedly from the initial day 0, with average values of 29 and 6.5, respectively (Figure 2B); they then decreased during days 3–15, followed by a gradual increase, reaching 37 and 19.5, respectively, at the end of fermentation; the total number of volatile compounds finally peaked at day 60 (40.5). The number of alcohols reached the maximum at day 7 (11.5), and then remained generally stable, ranging from 11 to 11.5, until the end of fermentation. In addition, no significant changes were observed in the numbers of other volatile compounds such as esters throughout the fermentation process. During the fermentation process, the number of unique volatile compounds in FGs decreased, from 9 at the early fermentation stage (day 0) to 4 at day 3; the reduced compounds were mainly esters and acids, with esters accounting for a relatively large proportion (Figure 2C). The types of volatile compounds were the most abundant in the early fermentation stage, decreased in the middle stage, and increased again in the late stage; the newly added types of volatile compounds from the middle to the late stage were mainly esters (Figure 2B,D). Overall, among the several types of volatile compounds, the number of ester types exhibited the largest fluctuation.

### 3.3. Fungal Community α-Diversity Analysis

Following quality control of the raw sequences, alpha diversity analysis was performed using clean sequences that had been randomly normalized to 20,000 (Figure S1). The Ace and Chao1 indices, which were utilized to quantify the richness of the fungal community, displayed a similar trend, exhibiting an initial decline followed by a subsequent rise (Figure S1A,B). Specifically, the lowest values were observed on the 7th day of the fermentation process, with readings of 131.93 ± 58.03 for Ace and 134.19 ± 61.19 for Chao1. Conversely, the highest values were recorded on the 45th day, reaching 409.64 ± 35.24 for Ace and 317.44 ± 45.31 for Chao1, respectively. In general, the Simpson and Shannon indices of the samples displayed a similar pattern, with an initial decrease followed by a subsequent increase (Figure S1C,D). This indicated an increase in species diversity toward the end of fermentation. Both indices reached their minimum values (0.77 ± 0.01 and 2.8 ± 0.07) on the 3rd day and maximum values (0.97 ± 0.02 and 6.10 ± 0.66) on the 60th day of fermentation, respectively. Significant differences were observed (*p* < 0.05) between the values on the 0th, 3rd, 7th, and 25th days compared to those on the 60th day.

### 3.4. Fungal Community β-Diversity Analysis

Cluster analysis (Figure S2A) and principal component analysis (PCA) (Figure S2B) were performed on the microbial community contents in FGs at different fermentation time points. The results showed that the fungal communities underwent dynamic changes during fermentation, and all samples were clustered into 3 distinct categories based on the long-term fermentation time series. Among them, the fungal communities at 0–3 days of fermentation were closely clustered, indicating similar community structures in the early fermentation stage (Category 1); the fungal communities at 7–25 days of fermentation were clustered into Category 2; and those at 45–60 days of fermentation were clustered into Category 3. There was a significant correlation between the succession of fungal communities in FGs and fermentation time, with day 7 being the turning point between the early and middle fermentation stages, and day 45 being the boundary point between the middle and late fermentation stages. Thus, the dynamic changes in fungal communities were consistent with the three stages of temperature dynamic changes shown in Figure 1A, which verified the rationality of dividing the entire fermentation process into the early, middle and late stages. It also indicated that there was synergy among temperature changes, physicochemical properties, and changes in fungal microbial communities during CSFB fermentation.

### 3.5. Fungal Community Composition

#### 3.5.1. Relative Content and Dynamics at the Level of Fungal Community Clades

**Figure 3:**
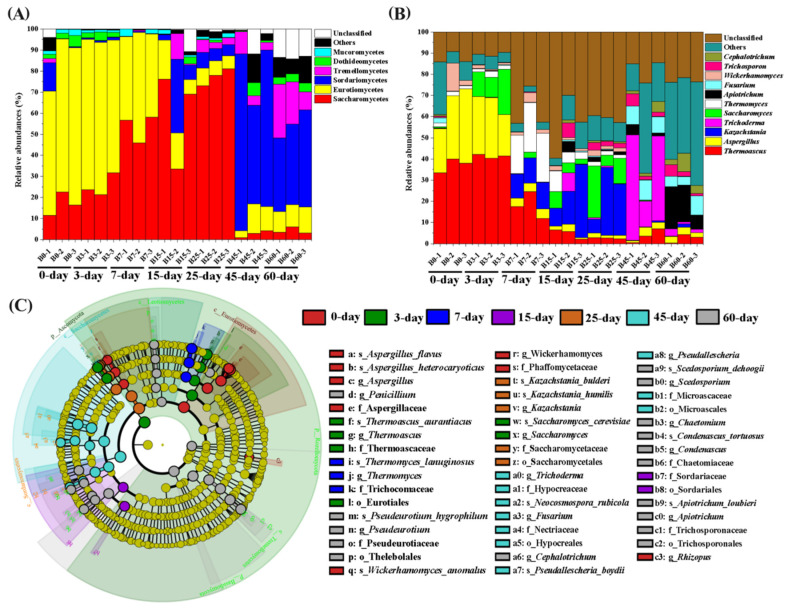
Fungal community composition during CSFB fermentation in different periods. Dynamic changes in fungi in FGs during CSFB fermentation (**A**). Dynamic change in dominant fungi in FGs during CSFB fermentation (**B**). Prediction of gene phenotype of FG fungi during CSFB fermentation (**C**).

Based on the analysis results of all samples, a total of 9 fungal phyla were identified, with significant differences in their relative abundances (Figure 3A). Among them, Ascomycota had the highest average relative abundance (89.13%), being the dominant phylum; Basidiomycota (6.03%) and Mortierellomycota (1.10%) were the second and third dominant phyla, respectively. Three dominant phyla reached the dominance threshold (relative abundance ≥ 1%), namely Ascomycota, Basidiomycota, and Mortierellomycota, accounting for 96.26% of all samples in total. The remaining 6 phyla, including unclassified fungal groups (1.44%), Mucoromycota (0.99%), and Chytridiomycota (0.61%), accounted for less than 4% in total. From a temporal perspective, with the extension of fermentation time, the number of dominant fungal phyla in FGs showed a gradual increasing trend: 2 phyla in the early fermentation stage, 2–4 phyla in the middle stage, and 4–5 phyla in the late stage, peaking at 5 phyla on day 60. As a persistent dominant phylum, Ascomycota exhibited a two-stage variation pattern in its abundance: it increased significantly in the early fermentation stage (0–3 days), reaching 98.21% on day 3 and peaking at 98.46% on day 7; then it decreased steadily to 93.65% on day 25 in the middle fermentation stage, followed by a sharp decline in the late stage, dropping to the minimum value of 65.63% on day 60. In contrast to Ascomycota, Basidiomycota showed an overall increasing trend: its relative abundance surged to 0.55% on day 3, decreased to 0.41% on day 7, and fluctuated in the middle stage (peaking at 0.53% on day 25); it proliferated explosively in the late fermentation stage, reaching the maximum value of 21.87% on day 60, which was 40 times higher than that on day 3. Among the remaining 7 phyla, except for Mucoromycota and Olpidiomycota, the relative abundances of the other phyla showed a continuous increasing trend, indicating that the diversity of fungal groups gradually increased in the late fermentation stage. Comprehensive analysis of the fungal communities in CSFB-FGs revealed that Ascomycota, Basidiomycota, Mortierellomycota, and unclassified fungal groups constituted the main body of the fungal communities. Ascomycota remained dominant throughout the entire fermentation process, with an average relative abundance exceeding 89% in the early stage; Basidiomycota and unclassified fungal groups became co-dominant groups in the middle fermentation stage (7–25 days), accounting for 20–25% in total; Mortierellomycota proliferated significantly in the late fermentation stage, increasing gradually from day 25 and becoming a dominant phylum by day 45, with a relative abundance of 15–20%. The above results indicated that the fungal communities in CSFB-FGs exhibited significant temporal dynamic succession characteristics, with the diversity of groups gradually increasing with the fermentation process. Core phyla such as Ascomycota, Basidiomycota, and Mortierellomycota sequentially dominated the community structure in specific fermentation stages.

#### 3.5.2. Relative Content and Dynamics of Fungal Communities at the Genus Level

A total of 195 fungal genera were identified in 21 FG samples, among which 12 dominant genera (relative abundance ≥ 1%) accounted for 61.17% of the total abundance of each sample (Figure 3B). The average relative abundances of these genera were as follows: *Thermoascus* (16.21%), *Aspergillus* (10.08%), *Kazachstania* (9.57%), *Thermomyces* (4.77%), *Trichoderma* (4.62%), *Saccharomyces* (4.17%), *Fusarium* (2.32%), *Apiotrichum* (2.30%), *Wickerhamomyces* (1.17%), *Cephalotrichum* (1.53%), *Trichosporon* (1.36%), and unclassified genera. These dominant genera belonged to 6 orders, concentrated in the classes Saccharomycetes, Eurotiomycetes, and Sordariomycetes. Taking *Thermoascus* and *Aspergillus* as examples, the average relative abundance of *Thermoascus* exhibited a trend of “first increasing, then decreasing, and slightly increasing again”: it reached the highest average relative abundance of 40.83% in the early fermentation stage (0–3 days); decreased significantly in the middle fermentation stage, dropping to the minimum value of 2.48% on day 25; and slightly rebounded in the late fermentation stage, reaching 3.68% on day 60. The continuous decline in the abundance of *Thermoascus* during fermentation may be related to the fermentation temperature not reaching its optimal growth temperature and the gradual consumption of nutrients. The average relative abundance of *Aspergillus* showed a trend of “first decreasing and then increasing”: it decreased continuously from the early to the middle fermentation stage, dropping to the minimum value of 1.39% on day 25; and slightly increased in the late fermentation stage, reaching 2.70% on day 60. *Aspergillus* remained a dominant fungal genus throughout the entire fermentation process, and the dynamic changes in its abundance may be associated with the consumption of nutrients and oxygen as well as the accumulation of metabolic products. The average relative abundances of *Thermomyces*, *Wickerhamomyces*, and *Alternaria* showed a downward trend at the end of fermentation, while those of *Kazachstania*, *Trichoderma*, *Saccharomyces*, *Fusarium*, *Apiotrichum*, *Cephalotrichum*, and *Trichosporon* exhibited an upward trend at the end of fermentation. These dominant genera exhibited genus-specific temporal dynamic changes during fermentation, which are speculated to be related to changes in factors such as temperature, nutrient availability, and oxygen content during the fermentation process.

#### 3.5.3. Prediction of Fungal Phenotypes

Using FUNGuild for phenotypic prediction analyses of operational taxonomic units (OTUs) from 21 samples, the resulting phenotypic prediction plots (Figure 3C) categorized the fungi into 16 functional groups based on their trophic modes. The most prevalent group was Undefined Saprotrophs, accounting for 71.50% of the fungi. Other significant groups included Animal Pathogens (16.16%), Fecal Saprotrophs (5.02%), Plant Pathogens (2.07%), and Animal Endosymbionts (1.37%). Smaller percentages were observed for Ectomycorrhizal (0.75%), Soil Saprotrophs (0.72%), Endophytes (0.59%), Fungal Parasites (0.52%), Litter Saprotrophs (0.35%), Arbuscular Mycorrhizal (0.27%), Wood Saprotrophs (0.24%), Animal Parasites (0.17%), Ericoid Mycorrhizal (0.17%), Algal Parasites (0.07%), and Leaf Saprotrophs (0.02%). These findings provide insights into the trophic strategies and functional roles of the fungal communities within the samples. The results show that the average relative abundance of Undefined Saprotrophs and Animal Pathogens was higher, accounting for a total of 87.66% of the OTUs. The overall trend was decreasing, with Undefined Saprotrophs reaching a peak on day 7, having an average relative abundance of 89.01%, while Animal Pathogens were highest at day 0, with an average relative abundance of 35.58%.

### 3.6. Factors Influencing Community Structure of Fungi in FGs

#### 3.6.1. Correlation Between Physicochemical Factors and Fungi

**Figure 4:**
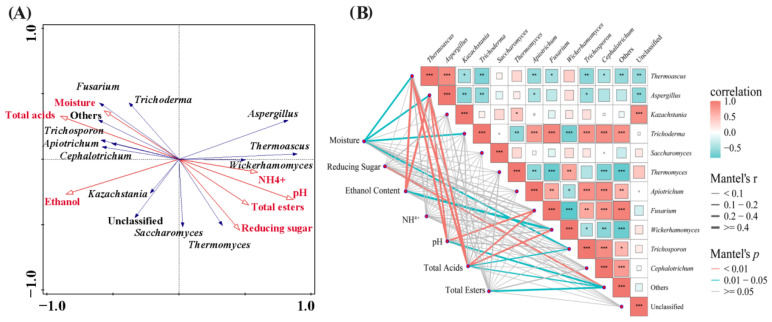
Correlation analysis between fungal community structure and physicochemical properties in FGs Redundancy analysis (**A**) and Mantel test of physicochemical factors and 12 dominant fungi genera (**B**). Note: *, **, and *** indicate significant correlations at the levels of *p* < 0.05, *p* < 0.01, and *p* < 0.001 based on the Mantel test, respectively.

RDA was employed to investigate the relationship between the physicochemical properties of FGs and the succession of 12 dominant fungal genera (Figure 4A). The results showed that the first axis of RDA explained 51.22% of the variation, and the cumulative contribution rate of the first two axes reached 65.22% of the total inertia. The fungal microbial communities were mainly concentrated along the first axis, indicating that the physicochemical properties of FGs played an important role in shaping the fungal community structure. The distribution patterns of the 12 dominant fungal genera were clearly distinguishable: 7 genera including *Trichoderma* and *Fusarium* were mainly located on the left side of the central vertical line of the first axis, showing positive correlations with TA, Et and moisture content, and negative correlations with the content of NH_4_^+^, pH value, RS and TE, indicating that these 7 fungal genera were acid-tolerant and alcohol-tolerant microorganisms; the remaining fungal genera including *Thermoascus* and *Aspergillus* were distributed on the right side of the central vertical line of the first axis, showing positive correlations with the content of NH_4_^+^, pH value, RS and TE, and negative correlations with TA, Et and moisture content. To further clarify the specific effects of various physicochemical properties on the fungal communities, Mantel tests were conducted between physicochemical factors and the 12 dominant fungal genera in this study (Figure 4B). The results showed that TA had the most significant effect on the fungal communities, explaining 57.40% of the variation; pH value (9.90%), moisture (8.10%), Et (8.00%), RS (7.50%), TE (1.60%) and NH_4_^+^ (0.60%) also contributed to the variation to a certain extent, but the influence degree was relatively weak. Notably, the effect of total acid on fungal microbial communities was significant (*p* < 0.05), while the effects of other physicochemical factors were not statistically significant (*p* > 0.05). In summary, the succession of dominant fungal communities in FGs during different fermentation periods was mainly affected by the content of TA, pH value, moisture, RS and Et, among which the effect of TA was the most prominent.

#### 3.6.2. Fungal Correlation Network Analysis of FGs

**Figure 5:**
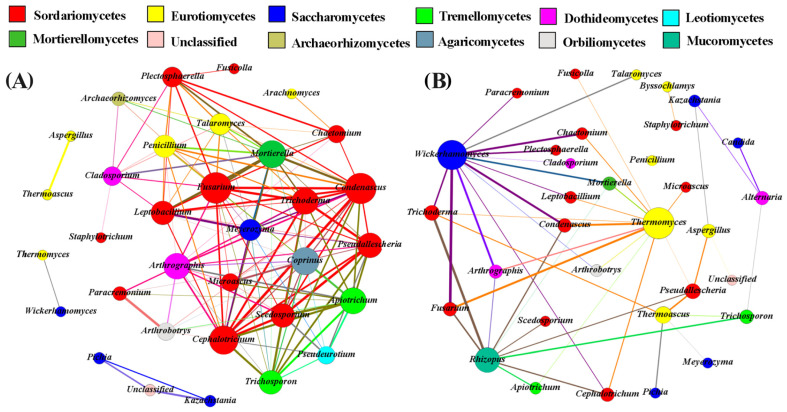
According to the correlation analysis, the cooccurrence and negative networks of genera with an average relative abundance of the more than 0.1% of FGs, the connections (i.e., edges) indicated a statistically significant (*p* < 0.01) strong positive (**A**), or negative correlation (**B**) (Spearman’s |*ρ*| > 0.7), and different classifications were represented by different colors. The size of each node is proportional to the number of connections, and the thickness of each edge between two nodes is proportional to the value of |*ρ*|.

To elucidate the complex intergeneric interactions among fungal genera in FGs, genera with an average relative abundance greater than 0.1% were selected for network analysis based on the results of Spearman’s correlation analysis (|*ρ*| > 0.7, *p*< 0.01), with each genus treated as a network node (Figure 5).

The positive correlation network at the genus level consisted of 102 edges and 27 nodes (Figure 5A), and 85.19% of the positive correlations occurred between fungal microorganisms of different classes, indicating high interclass connectivity. Specifically, Sordariomycetes accounted for 37.04% of the total positive correlations, followed by Eurotiomycetes (18.52%), Tremellomycetes (11.11%), Saccharomycetes (11.11%), and Dothideomycetes (7.41%). This distribution characteristic suggests that the interactions between fungal microorganisms of different classes play an important role in the shaping and succession of community structure. Nine key core microbial taxa were identified in the positive correlation network, defined as nodes with positive correlations with at least 8 other genera, including *Mortierella*, *Trichoderma*, *Cephalotrichum*, *Scedosporium*, *Apiotrichum*, *Condenascus*, *Fusarium*, *Arthrographis*, and *Trichosporon*. These core microbial taxa were distributed across 4 classes: Sordariomycetes (5), Tremellomycetes (2), Dothideomycetes (1), and Mortierellomycetes (1). They may act as keystone species to promote the interactions and connectivity within the fungal community. The negative correlation network comprised 19 edges and 22 nodes (Figure 5B). Similarly to the positive correlation network, 81.83% of the negative correlations occurred between fungal microorganisms of different classes. Sordariomycetes accounted for 31.82% of the negative correlations, followed by Saccharomycetes (22.73%), Dothideomycetes (13.64%), and Eurotiomycetes (13.64%). Notably, only one core microbial taxon–Wickerhamomyces–was identified in the negative correlation network, which was connected to 9 other nodes, indicating that it may play a core regulatory role in the negative interaction network. In summary, Mortierella, Trichoderma, Cephalotrichum, Scedosporium, Apiotrichum, Condenascus, Fusarium, Arthrographis, Trichosporon and Wickerhamomyces are key nodes in the fungal correlation network and play important roles in maintaining network connectivity.

### 3.7. Correlation Between Fungal Microorganisms and the Dynamics of Volatile Compounds in FGs

**Figure 6:**
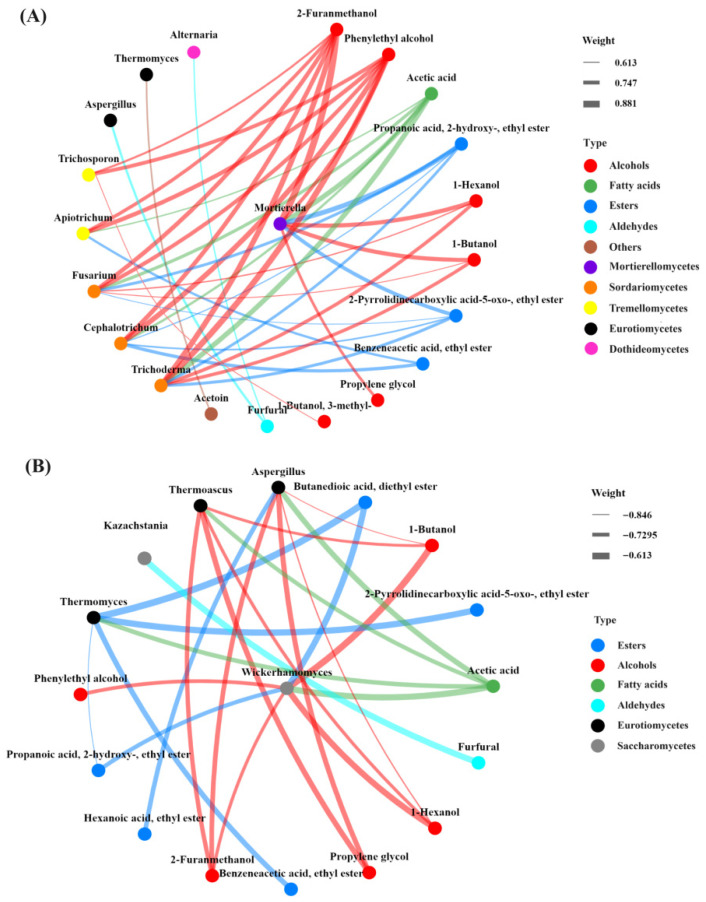
The positive (**A**) and negative network relationships (**B**) between 12 dominant genera of FGs and 12 flavor compounds were analyzed based on Spearman correlation.

To clarify the correlations between fungal communities and volatile compounds during CSFB fermentation, based on the relative contents of fungal communities at the genus level, their dynamic changes, and the results of fungal correlation network analysis in FGs, Spearman’s correlation coefficients between these fungal genera (including 12 dominant fungal genera and 9 key nodes in the fungal correlation network) and 47 volatile compounds were calculated, and a network diagram was constructed (Figure 6).

Among them, 12 fungal genera were correlated with at least one of the 12 volatile compounds, and these genera belonged to Dothideomycetes, Eurotiomycetes, Mortierellomycetes, Sordariomycetes, Tremellomycetes, and Saccharomycetes. The aforementioned 12 volatile compounds included 6 alcohols, 4 esters, 1 phenol, and 1 other compound. Specifically, 9 fungal genera showed positive correlations with at least one of the 12 compounds (Figure 6A). Among these 9 genera, 45.45% were positively correlated with alcohols, 36.36% with esters, 9.09% with phenols, and 9.10% with other compounds. Positive correlations were mainly concentrated in four genera: *Mortierella*, *Cephalotrichum*, *Fusarium* and *Trichoderma*, among which *Mortierella* exhibited the most prominent positive correlation associations. In terms of negative correlations, 5 fungal genera were negatively correlated with at least one of the 12 volatile compounds (Figure 6B). Among them, 57.14% were negatively correlated with alcohols, 28.57% with esters, and 14.29% with phenols. Negative correlations were mainly concentrated in three genera: *Thermomyces*, *Aspergillus*, and *Wickerhamomyces*, among which *Wickerhamomyces* showed the most significant negative correlation associations.

## 4. Discussion

As mentioned in the introduction, during the long-cycle fermentation process of CSFB brewing, FGs serve as the core component of the solid-state fermentation, harboring an extremely complex microbial flora system. Throughout the long-cycle fermentation of CSFB, the microbial communities in FGs undergo continuous succession, and the physicochemical properties as well as the formation of flavor substances exhibit complexity and diversity. Fungi are the core and irreplaceable microbial group in FGs, and their role runs through the entire Baijiu fermentation process, determining the flavor characteristics of the Baijiu. Therefore, it is of great significance to study the succession patterns of fungal communities in FGs, explore their influencing factors, and analyze their relationships with volatile compounds. This will provide theoretical guidance for the production of CSFB.

Based on the dynamic changes in fermentation temperature and physicochemical properties (Figure 1), the entire fermentation process can be divided into three distinct stages: the early stage (0–7 days), the middle stage (7–25 days), and the late stage (45–60 days). *Thermoascus*, *Aspergillus*, and *Saccharomyces* entered a rapid proliferation period, which is basically consistent with the changes in dominant microbial genera (Figure 3B). Combined with the results of Et and RS (Figure 1B), it is analyzed that in the early fermentation stage, a large number of *Thermoascus* enriched during the high-temperature starter-making stage were introduced into FGs, assisting *Aspergillus* in degrading starch into RS and promoting ethanol synthesis under the synergistic action of microorganisms such as *Saccharomyces* [21]. During the middle fermentation stage, the dynamic changes in the physicochemical properties of FGs, such as moisture content, ethanol content, and total acid content, were largely consistent with the findings reported by Liu et al. in their study on the FGs of Chinese Tao-rong-flavor Baijiu. Such similar results observed in FGs of different Baijiu flavor types also confirm the universal rule in the solid-state fermentation of Chinese distilled Baijiu: microbial metabolism drives the evolution of the physicochemical environment, and the physicochemical environment selects functional microbial communities [22]. Combined with the heatmap of ester content changes in Figure 2A, core esters synthesized in the early stage (e.g., ethyl butyrate and ethyl caproate) were hydrolyzed, resulting in decreased contents. It is speculated that the synergistic changes in environmental conditions, microbial community succession, and substrate supply of FGs in the middle stage led to the hydrolysis rate of esters exceeding the synthesis rate. In the middle fermentation stage, microbial groups such as *Lactobacillus* proliferated and secreted extracellular esterases, preferentially hydrolyzing short-chain esters [22]. In addition, esterification reactions require a microaerobic environment, but FGs in the middle stage became compact due to microbial proliferation, forming a strictly anaerobic environment inside FGs, which may inhibit microaerobic-dependent esterification reactions [23]. In the late fermentation stage, the total amount of esters showed a decreasing trend, while the types of esters increased (Figure 2). Hu et al. also reported the same finding: the newly detected flavor substances in the late fermentation stage of CSFB were esters (e.g., ethyl caproate and ethyl lactate), and they considered that esterification reactions were prominent in this stage, referring to the late stage as the esterification period [24]. Combined with microbial diversity in Figure S1 and changes in dominant microorganisms in Figure 3A,B, microbial diversity increased in the late fermentation stage. Different microorganisms targeted the synthesis of specific esters, and the microenvironment was suitable for multiple esterification reactions; meanwhile, various esters gradually accumulated, ultimately leading to a significant increase in the types of esters. This is also the core reason why the flavor of CSFB gradually enriches with the extension of the fermentation cycle [25]. In addition, the results of dynamic changes in physicochemical properties (Figure 1B,C) and changes in flavor substances (Figure 2) also indicated that the middle fermentation stage is a critical period for RS utilization and product accumulation, which is consistent with previous reports [26,27]. The efficient utilization of RS in the middle fermentation stage not only accumulates direct products such as Et and TA but also provides key precursors for ester synthesis in the late stage.

During the fermentation of FGs for CSFB production, Ascomycota and Basidiomycota were the dominant phyla (with an average relative abundance exceeding 1%) (Figure 3A). Previous studies have indicated that Ascomycota is the dominant fungal phylum in the fermentation of FGs for CSFB, Maotai-flavor, and light-flavor Baijiu, with a relative abundance ranging from 96.57% to 99.99% [28,29]. This finding not only confirms the key role of Ascomycota in Baijiu fermentation but also reveals the commonalities of core microorganisms involved in the fermentation of Baijiu with different flavor types. The average relative abundance of Basidiomycota showed an opposite trend to that of Ascomycota. This finding suggests that there may be an inhibitory interaction between the growth of Ascomycota and Basidiomycota, which is consistent with previous research results [30]. From the results of dominant genera during CSFB fermentation, the dominant genera included *Thermoascus*, *Aspergillus*, *Kazachstania*, *Thermomyces*, *Trichoderma*, *Saccharomyces*, *Fusarium*, *Apiotrichum*, *Wickerhamomyces*, etc. (Figure 3B). High abundances of *Thermoascus*, *Aspergillus*, *Kazachstania*, *Trichosporon*, etc., have also been found in FGs for the fermentation of Feng-flavor Baijiu [31]. In Baijiu production, *Thermoascus* and *Thermomyces* mostly produce various thermostable enzymes such as cellulase, protease, and amylase, which decompose macromolecular substances such as polysaccharides and proteins in FGs, thereby promoting the growth and reproduction of microorganisms and the production of aroma-active substances [32]. *Aspergillus* can efficiently decompose macromolecular substances such as starch and proteins, and also significantly promote the synthesis of esters [16]. *Saccharomyces* plays a key role in Baijiu fermentation; in addition to metabolically producing a large amount of ethanol, it can also generate a variety of flavor compounds [33]. *Wickerhamomyces* can utilize FGs to produce a variety of flavor compounds, making an important contribution to the formation of the characteristic flavor.

The structure of eukaryotic microbial communities in FGs of CSFB at different fermentation time points was significantly affected by physicochemical factors (Figure 4) and microbial factors (Figure 5). In this study, 7 fungal genera were found to be positively correlated with TA, Et, and moisture content, and negatively correlated with NH_4_^+^, pH value, RS, and TE contents, while the remaining fungal genera were positively correlated with NH_4_^+^, pH value, RS, and TE contents. TA was found to have the most significant effect on fungal communities. The pH value of FGs is a major factor ensuring normal fermentation; an appropriate acidity level is essential for microbial growth and metabolism, and excessively high or low acidity will have adverse effects on microorganisms [10,34,35,36]. Li et al. found that the acidity of FGs drives the succession of microbial communities, leading to increases in phenethyl alcohol content and quality of both FGs and base Baijiu, and pH is the most important environmental factor controlling the succession of fungal communities [37]. Huang et al. reported that *Kazachstania* and *Wickerhamomyces* are the absolutely dominant genera during the fermentation of CSFB, and the acidity in FGs is a key factor affecting the succession of microbial community structure, which is basically consistent with the results of this study [27]. According to the research results in Figure 5, fungi such as *Mortierella*, *Trichoderma*, *Cephalotrichum*, *Scedosporium*, *Apiotrichum*, *Condenascus*, *Fusarium*, *Wallemia*, *Trichosporon*, and *Wickerhamomyces* are key nodes in the correlation network, and there is a high degree of overlap with the dominant genera identified earlier (Figure 3B). As the core of enzyme systems for raw material degradation (e.g., *Trichoderma*, *Cephalotrichum*, *Fusarium*), these genera can secrete specific hydrolases to decompose macromolecules such as starch and cellulose; alternatively, they undertake the core functions of alcohol fermentation and characteristic ester synthesis (e.g., *Wickerhamomyces*, *Trichosporon*, *Apiotrichum*, *Condenascus*); or they maintain metabolic activity in the fermentation environment by virtue of characteristics such as high osmotic pressure tolerance and high temperature tolerance (e.g., *Wallemia*, *Scedosporium*). Meanwhile, they build bridges for material and information exchange in microbial communities through interactive relationships such as symbiosis and antagonism, forming a functional consortium with complementary enzyme systems and metabolic synergy. This consortium runs through the entire fermentation stage of CSFB from raw material degradation to flavor formation, collectively ensuring the stability of the fermentation process and the formation of characteristic flavors [38].

In the correlation analysis of dominant genera, key nodes and flavor substances in FGs, we found that there were complex correlations between fungal genera and flavor substances. These intricate biological relationships constitute the fundamental biological regulatory mechanisms of the CSFB fermentation process, and also form the core component of CSFB fermentation kinetics, flavor formation, and the regulatory mechanisms of the fermentation process. Previous studies have indicated that yeasts dominate the microbial network, and the genus *Scedosporium*, acting as a core hub, is crucial for the connectivity of the entire network [39]. Notably, most of the dominant genera in the early stage were predominantly correlated with 1-butanol and 1-hexanol. In addition, previous studies have also found that *Thermomyces* was significantly negatively correlated with 1-butanol [40]. Meanwhile, 1-butanol and 1-hexanol have been confirmed to be important compounds affecting the unique flavor of Baijiu, highlighting their significance in the complex flavor characteristics of this traditional alcoholic beverage [37]. *Trichoderma*, *Fusarium* and *Mortierella* were all significantly positively correlated with 1-butanol and 1-hexanol (*p* ≤ 0.05), suggesting that they may contribute to the flavor characteristics of the product. In contrast, *Wickerhamomyces* and *Aspergillus* were significantly negatively correlated with these two compounds (*p* ≤ 0.05), implying that they may exert an inhibitory effect on the accumulation of these two compounds. Furthermore, studies have demonstrated that *Aspergillus* can biosynthesize esters using acids and alcohols as substrates [41,42,43]. Based on these findings, it can be inferred that different microorganisms are capable of producing the same volatile compounds through their respective distinct metabolic pathways. Additionally, microorganisms of a single genus can metabolize to generate a variety of volatile compounds, which indicates that there exist complex interactions between microbial species and volatile compounds [44]. This study identified the key fungal genera that are significantly correlated with the dynamic changes in volatile compounds in fermented FGs during CSFB production.

## 5. Conclusions

In summary, this study comprehensively revealed the core characteristics of fungal communities and related influencing factors in CSFB-FGs: the fungal communities exhibited distinct stage-wise succession characteristics during fermentation; Ascomycota was the absolutely dominant fungal phylum throughout the entire process and had an inhibitory interaction with Basidiomycota; genera such as *Thermoascus*, *Aspergillus*, and *Wickerhamomyces* were the core dominant genera during the whole fermentation process. Among physicochemical factors, total acid exerted the most significant regulatory effect on fungal community structure, while pH value drove community succession by affecting nutrient dissociation, microbial cell membrane functions, etc. The middle fermentation stage was a critical period for reducing sugar utilization and the accumulation of products such as ethanol and total acids, and its metabolites provided precursors for ester synthesis in the late stage. Fungal genera including *Mortierella* and *Trichoderma* were key nodes in the correlation network and highly overlapped with dominant genera; these fungi may form a functional whole with complementary enzyme systems and metabolic synergy through interactions such as symbiosis and antagonism. The association between fungal communities and volatile flavor substances showed significant specificity: genera such as *Trichoderma* and *Fusarium* could promote the accumulation of key flavor substances including 1-butanol and 1-hexanol, while *Wickerhamomyces* and *Aspergillus* had an inhibitory effect on the accumulation of such substances. Moreover, a complex interaction pattern was observed between fungi and flavor substances; the increased microbial diversity in the late fermentation stage further promoted an increase in the types of ester substances, ultimately enriching the Baijiu flavor. The results of this study provide theoretical support for the regulation of the CSFB fermentation process and the optimization of flavor quality. More importantly, the results indicate that there are potential and complex metabolic interactions among various species in FGs during long-term fermentation, suggesting that an in-depth exploration of these interactions can be a focus of future research.

## Data Availability

The sequencing data were submitted to the Sequence Read Archive (SRA) of the NCBI database under BioProject PRJNA1065102 (BioSample accession numbers SUB14153192). Other contributions presented in the study are included in the article/Supplementary Material, further inquiries can be directed to the corresponding authors.

## Supplementary Materials

The following supporting information can be downloaded at https://www.mdpi.com/article/10.3390/foods15030418/s1, Figure S1. Alpha diversity analysis of samples with clean sequences after raw sequence quality control. Abundance-based coverage estimator (A). Chao1 estimator of richness (B). Simpson index (C) and Shannon diversity index (D). Figure S2. PCA of content of microbial communities in FGs at different fermentation time-points. Clustering results of the FGs samples (A). Principal component analysis of fungal community content in FGs samples (B).

## Abbreviations

The following abbreviations are used in this manuscript:

CSFB	Chinese strong-flavor Baijiu
FGs	Fermented grains
TA	Total acid content
Et	Ethanol content
RS	Reducing sugars content
TE	Total esters content
RDA	Redundancy analysis
PCA	Principal component analysis
