# Supplementary material for: Illuminating the Patterns of Fungal Community Succession, Physicochemical Properties, Volatiles and Their Relationships in Fermented Grains for the Production of Chinese Strong-Flavor Baijiu
Source: Foods. 2026 Jan 23;15(3):418. doi: 10.3390/foods15030418 (PMC12897240; doi:10.3390/foods15030418)
Supplement: Supplementary file 1 [file foods-15-00418-s001.zip › foods-4086362-supplementary.pdf]

**Supplementary materials**

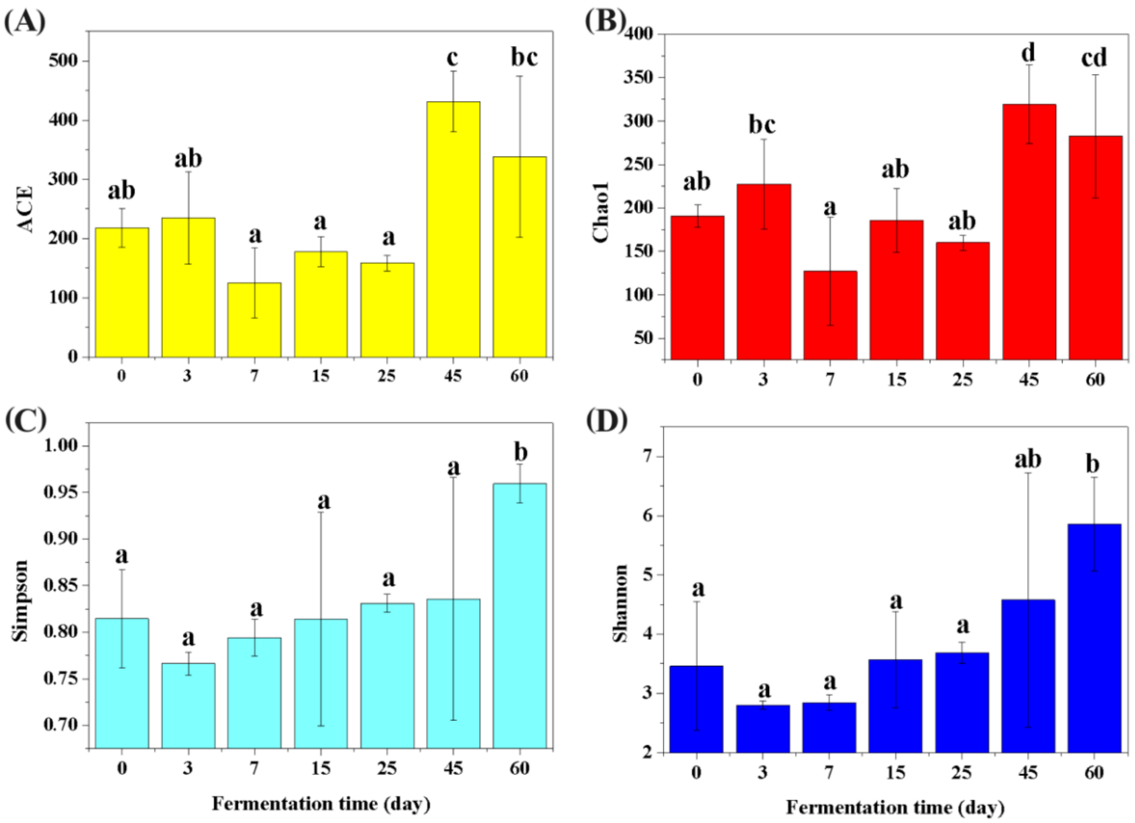


**Figure S1.** Alpha diversity analysis of samples with clean sequences after raw sequence quality control. Abundance-based coverage estimator (A). Chao1 estimator of richness (B). Simpson index (C) and Shannon diversity index (D).

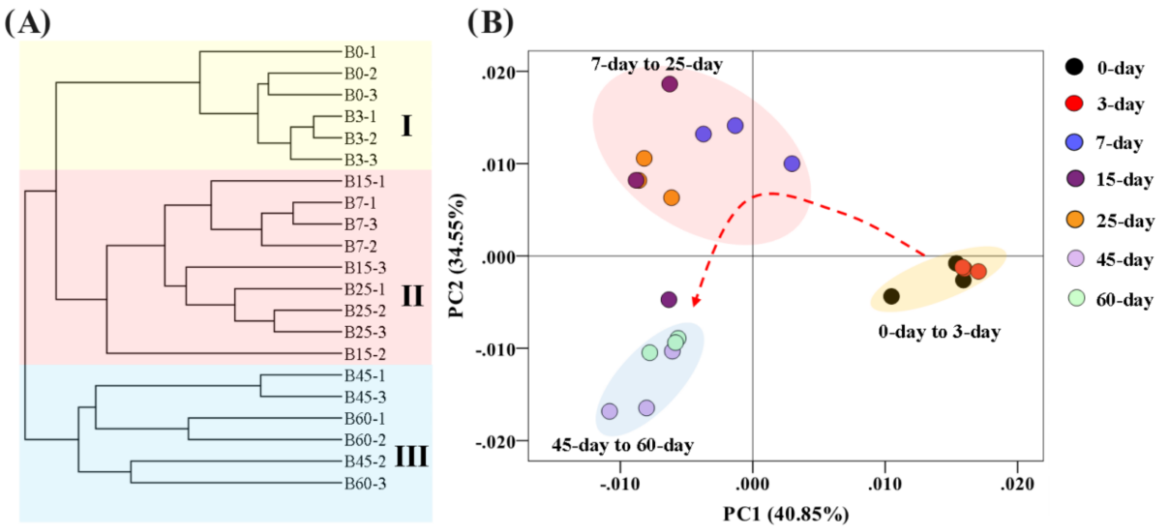


**Figure S2.** PCA of content of microbial communities in FGs at different fermentation time-points. Clustering results of the FGs samples (A). Principal component analysis of fungal community content in FGs samples (B).
